# Supplementary material for: Combining occurrence and abundance distribution models for the conservation of the Great Bustard
Source: PeerJ. 2017 Dec 13;5:e4160. doi: 10.7717/peerj.4160 (PMC5732545; doi:10.7717/peerj.4160)

Appendix 1 Six regression algorithms (TreeNet, Random Forest, CART, MARS, Regression and CART Ensemble in SPM7) to predict abundance (prediction abundance) from observed abundance data.

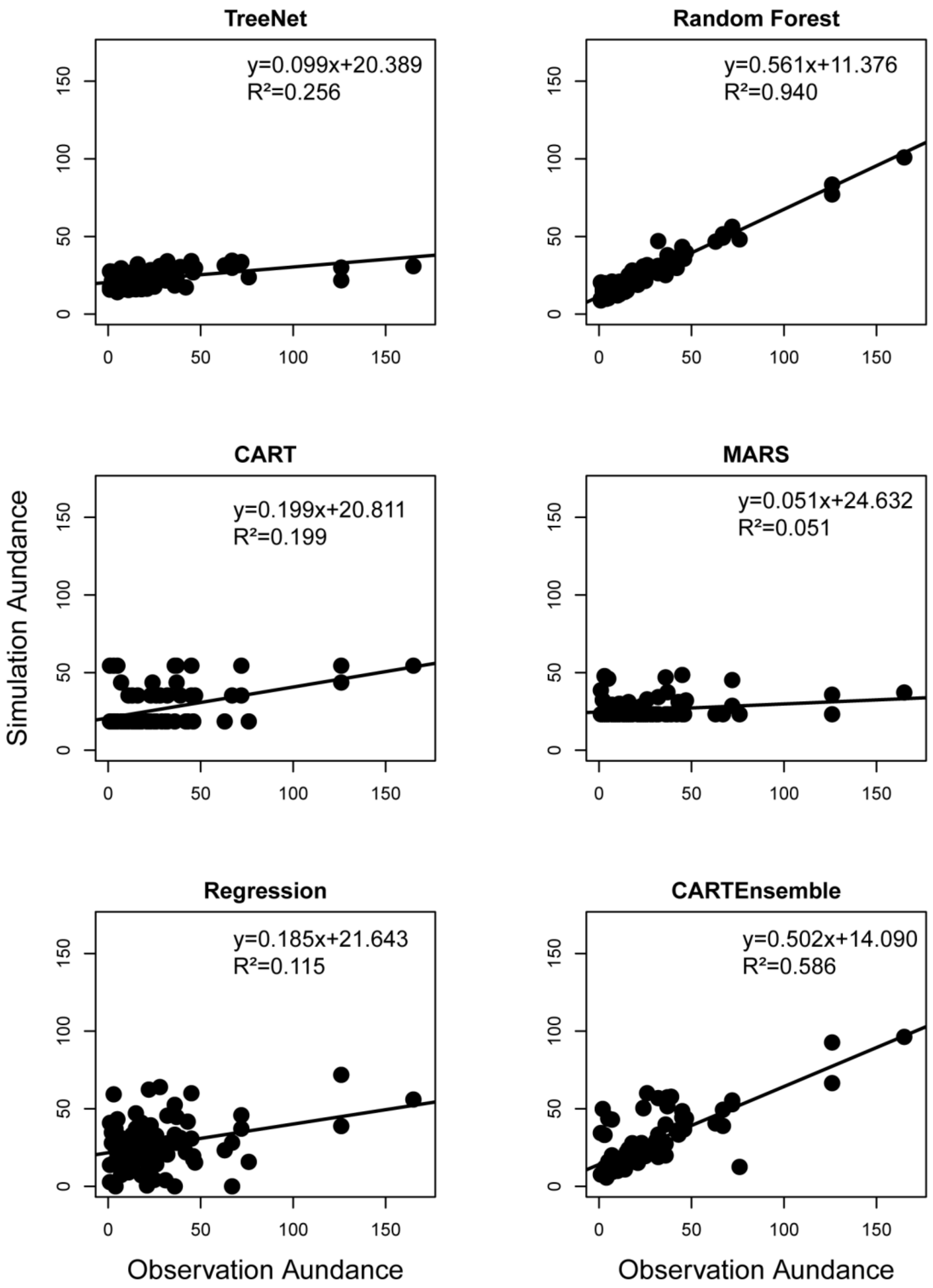

Supplement: Appendix S1 [file peerj-05-4160-s001.pdf]
