# Supplementary figures and images for: Combining occurrence and abundance distribution models for the conservation of the Great Bustard
Source: PeerJ. 2017 Dec 13;5:e4160. doi: 10.7717/peerj.4160 (PMC5732545; doi:10.7717/peerj.4160)

## Appendix 2 partial dependence plot for occurrence model (SDM) and abundance model (SAM)

### SDM

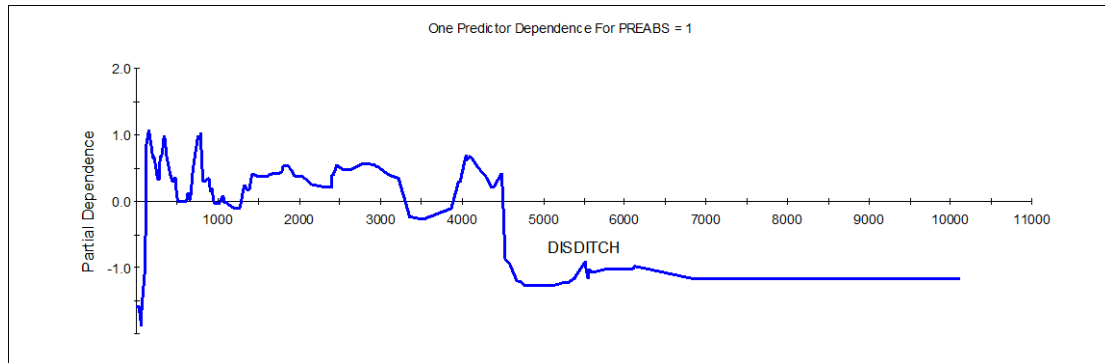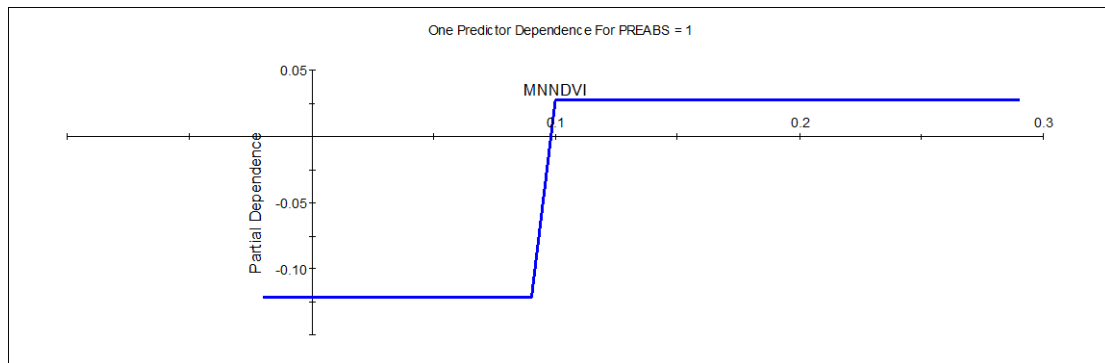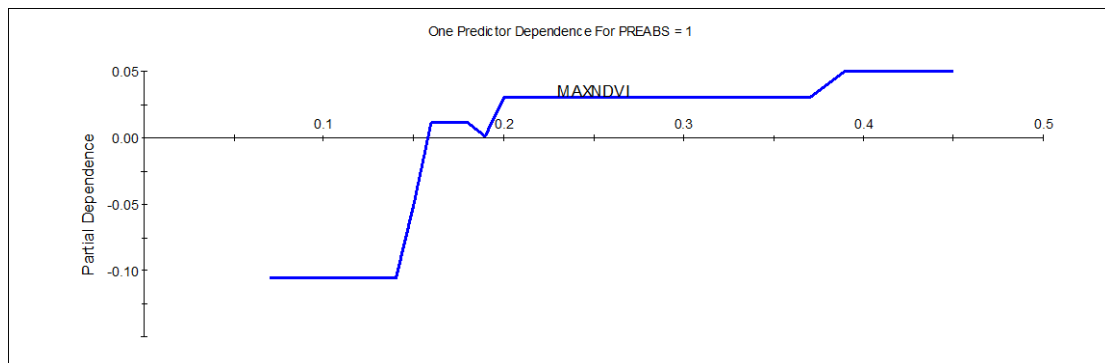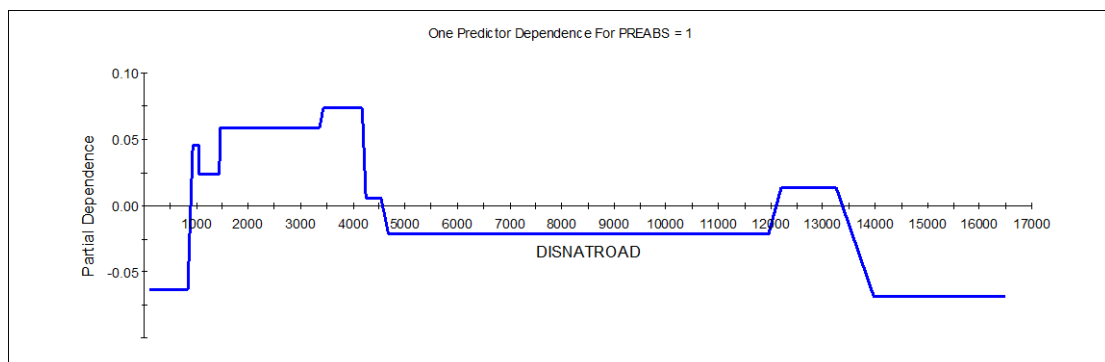

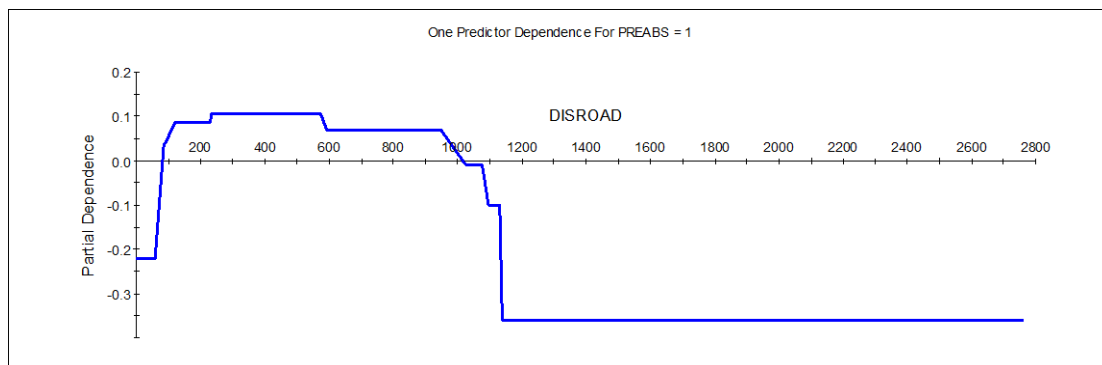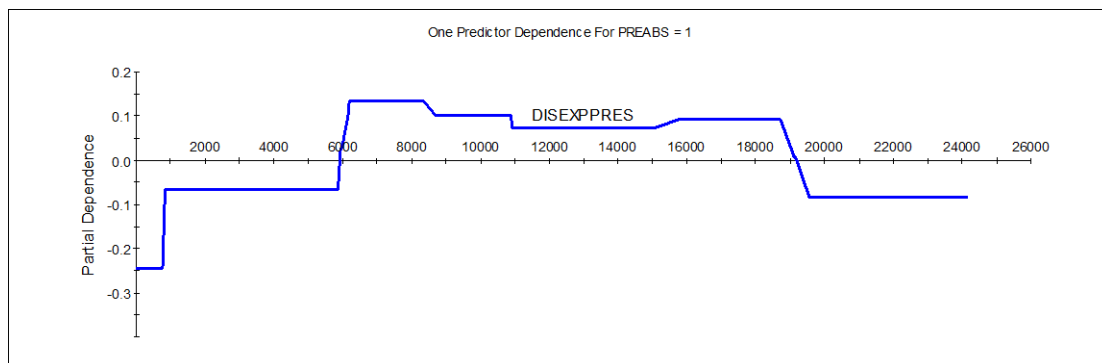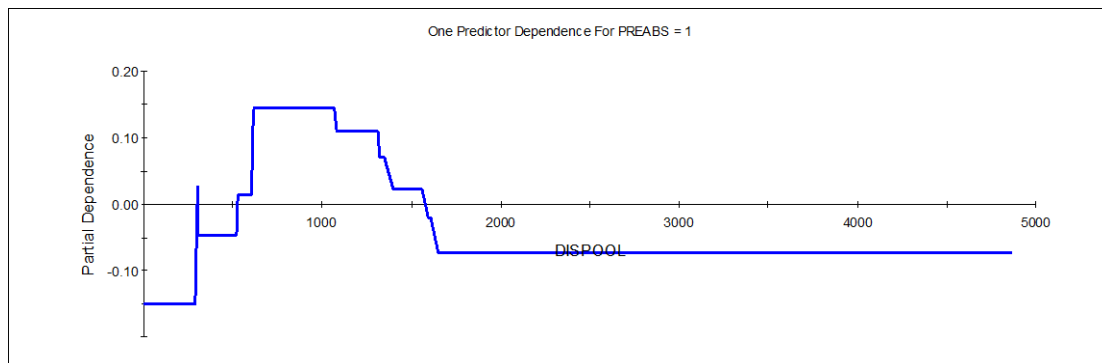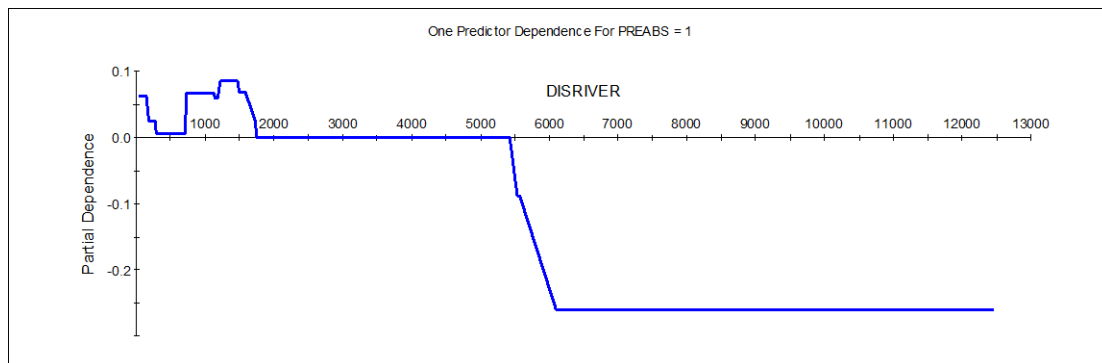

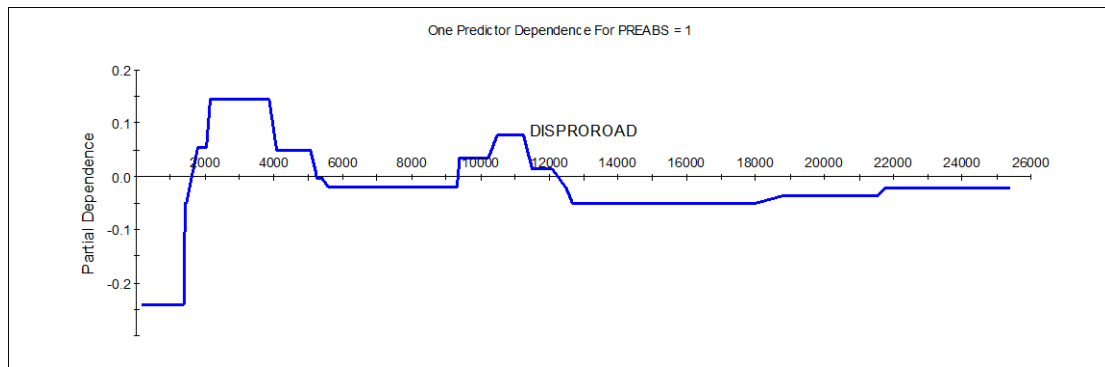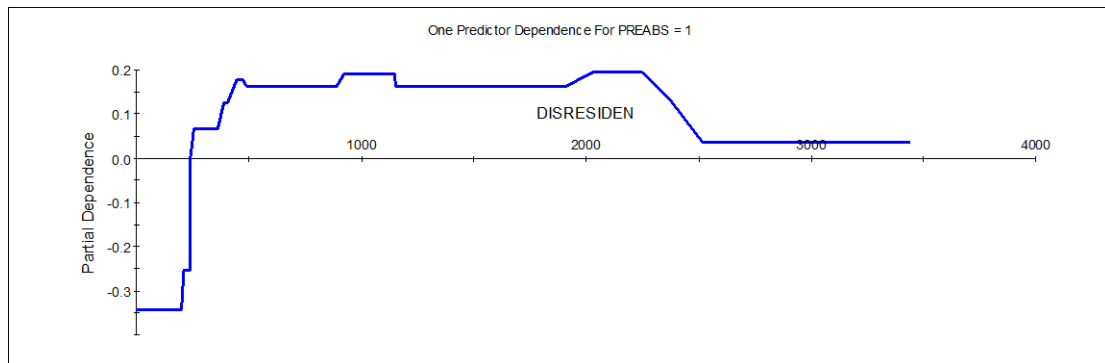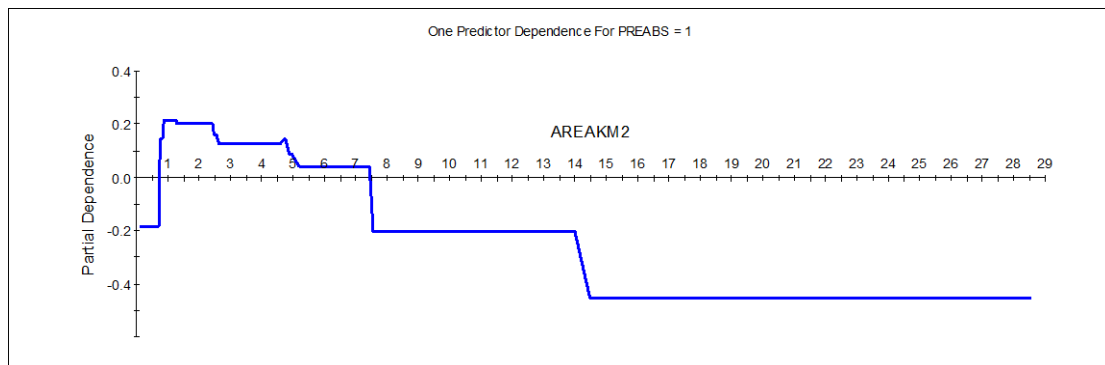

## SAM

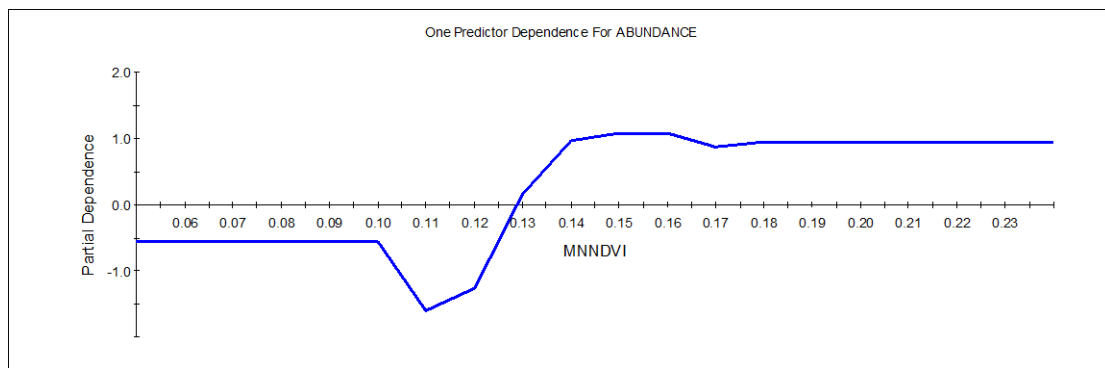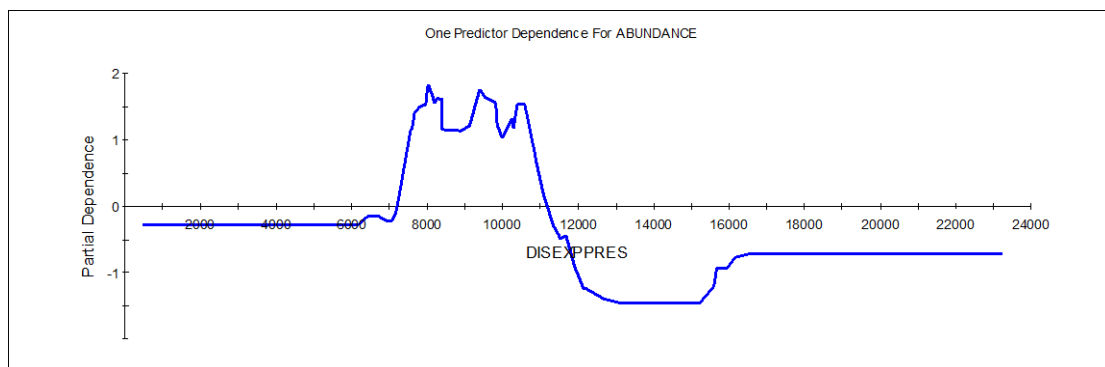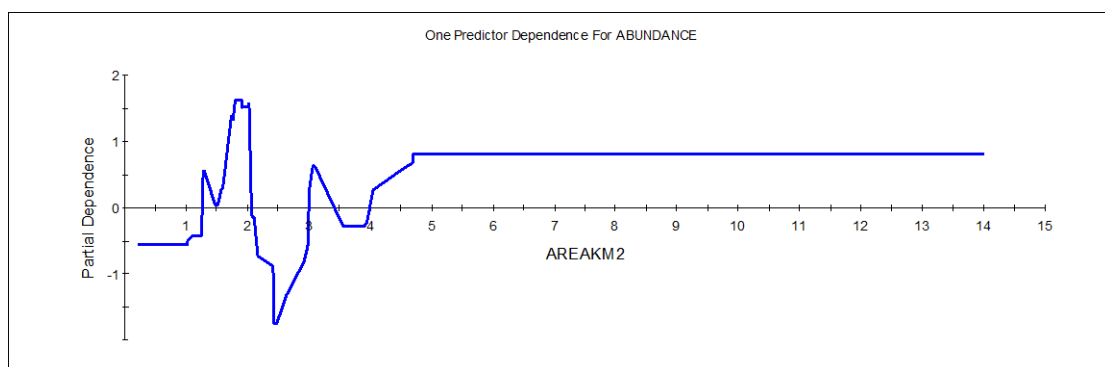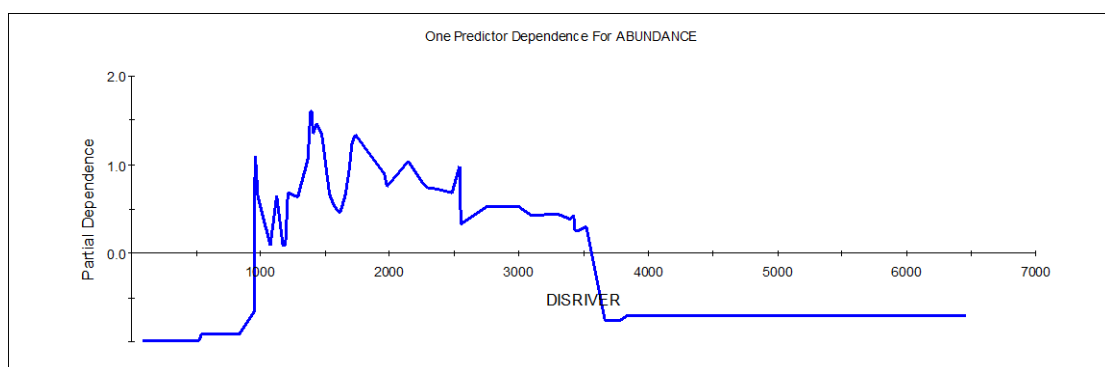

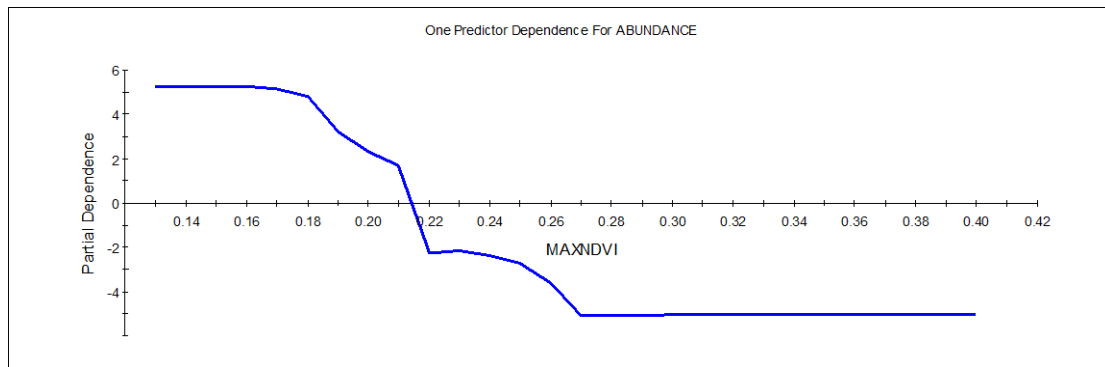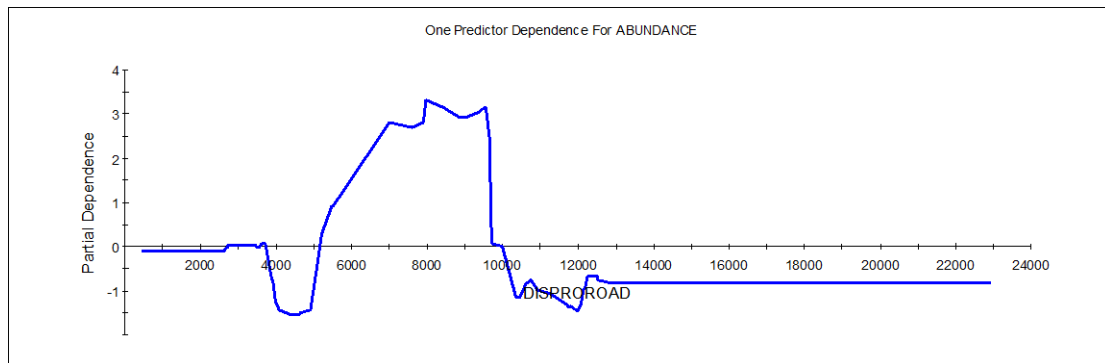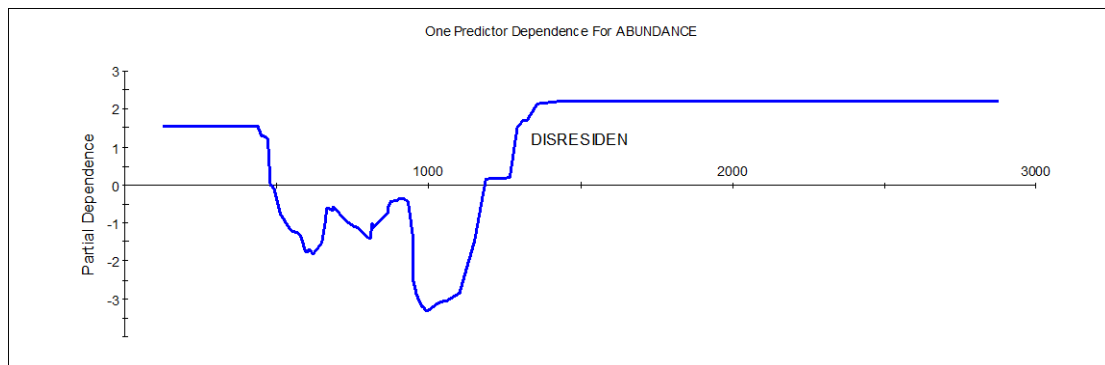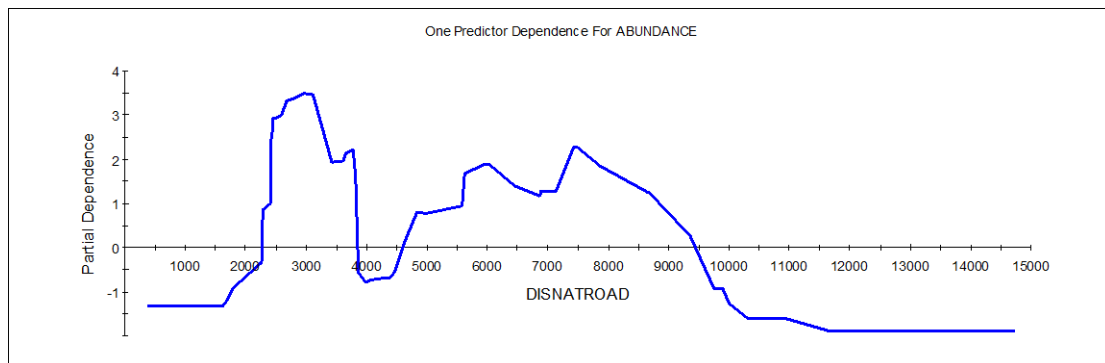

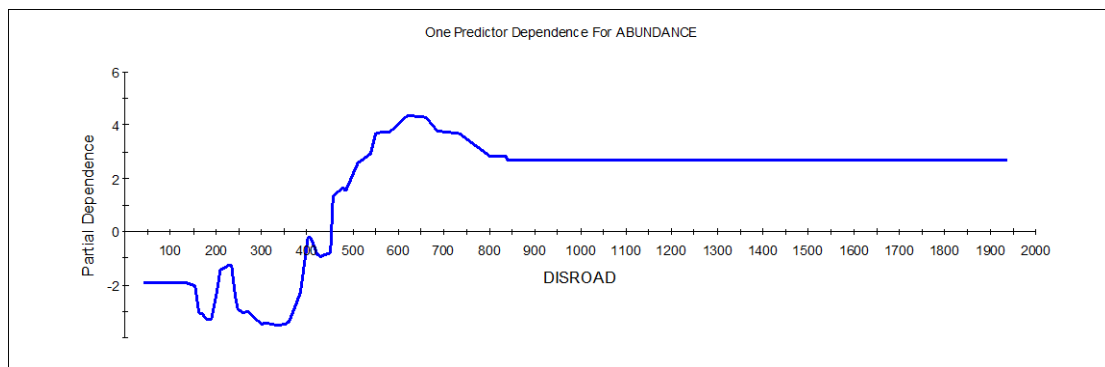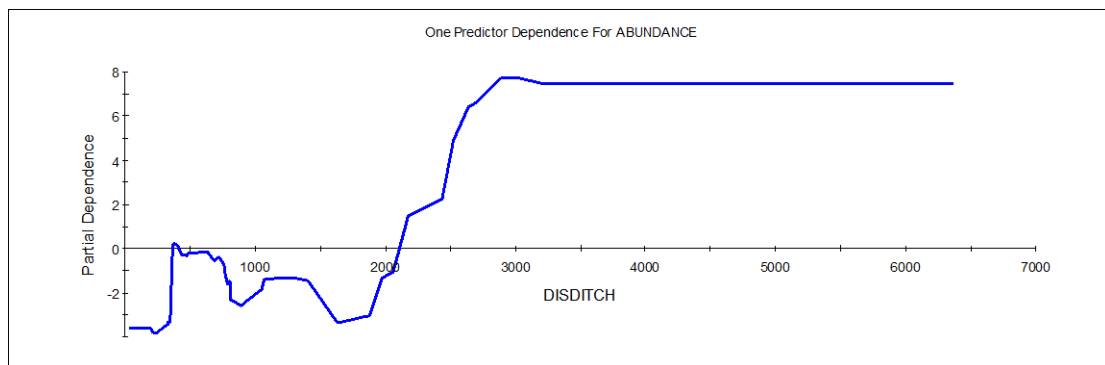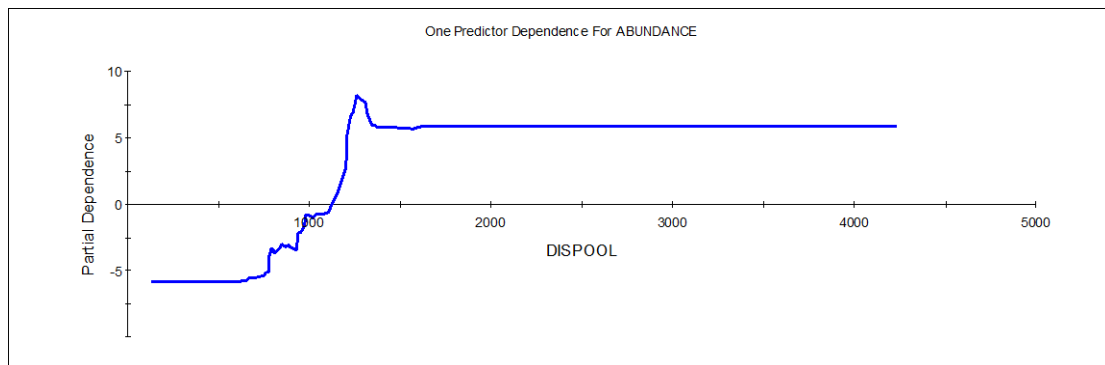

Supplement: Appendix S2 [file peerj-05-4160-s002.pdf]
